# Evidence for Intermolecular Interactions between the Intracellular Domains of the Arabidopsis Receptor-Like Kinase ACR4, Its Homologs and the Wox5 Transcription Factor

**DOI:** 10.1371/journal.pone.0118861

**Published:** 2015-03-10

**Authors:** Matthew R. Meyer, Shweta Shah, J. Zhang, Henry Rohrs, A. Gururaj Rao

**Affiliations:** 1 Roy J. Carver Department of Biochemistry, Biophysics and Molecular Biology, Iowa State University, Ames, Iowa 50011, United States of America; 2 Department of Medicine, Washington University School of Medicine, 660 S. Euclid Ave, St. Louis, MO 63130, United States of America; 3 NIH NCRR Center for Biomedical and Bio-Organic Mass Spectrometry, Dept. of Chemistry, Washington University, St. Louis, MO 63130, United States of America; University of Nottingham, UNITED KINGDOM

## Abstract

*Arabidopsis* CRINKLY4 (ACR4) is a receptor-like kinase (RLK) involved in the global development of the plant. The *Arabidopsis* genome encodes four homologs of ACR4 that contain sequence similarity and analogous architectural elements to ACR4, termed *Arabidopsis* CRINKLY4 Related (AtCRRs) proteins. Additionally, a signaling module has been previously proposed including a postulated peptide ligand, CLE40, the ACR4 RLK, and the WOX5 transcription factor that engage in a possible feedback mechanism controlling stem cell differentiation. However, little biochemical evidence is available to ascertain the molecular aspects of receptor heterodimerization and the role of phosphorylation in these interactions. Therefore, we have undertaken an investigation of the *in vitro* interactions between the intracellular domains (ICD) of ACR4, the CRRs and WOX5. We demonstrate that interaction can occur between ACR4 and all four CRRs in the unphosphorylated state. However, phosphorylation dependency is observed for the interaction between ACR4 and CRR3. Furthermore, sequence analysis of the ACR4 gene family has revealed a conserved ‘KDSAF’ motif that may be involved in protein-protein interactions among the receptor family. We demonstrate that peptides harboring this conserved motif in CRR3 and CRK1are able to bind to the ACR4 kinase domain. Our investigations also indicate that the ACR4 ICD can interact with and phosphorylate the transcription factor WOX5.

## Introduction

Mammalian systems have acquired signal transduction mechanisms via the use of receptor tyrosine kinases (RTK) to coordinate cellular processes such as proliferation, migration, differentiation, and cell-cycle control [1]. RTKs are comprised of an extracellular ligand binding domain, a single membrane spanning region, and an intracellular tyrosine kinase domain. The classical paradigm of RTK activation involves ligand binding to the extracellular domain and receptor homo- or heterodimerization through interactions among receptor subdomains [1–5].

Similar to RTKs in architecture, plant receptor-like kinases (RLKs) contain an extracellular ligand binding domain, a transmembrane helix, and an intracellular serine/threonine kinase domain and are involved in various plant signaling processes [6–9]. It is evident from structural and biochemical studies that mechanisms of RLK signaling are similar to RTKs where ligand binding leads to receptor activation and initiation of an intracellular signaling cascade. Furthermore, it has been demonstrated that RTKs can form homo- or heterodimeric receptor complexes that can elicit differential signaling cascades based on intracellular kinase autophosphorylation and recruitment of specific signaling molecules, as classically exemplified by the EGF receptor family [10–12]. Among RLKs, the heterodimeric interactions between BRI1 and BAK1 and its effects on downstream signaling are well documented [13–14].

*Arabidopsis* CRINKLY4 (ACR4) is a RLK involved in the proper growth and development of the plant [15–18]. We have recently reported on some significant *in vitro* biochemical properties of the ACR4 intracellular domain including its kinase activity, oligomerization properties, and the identification of at least 16 autophosphorylation sites encompassing all three subdomains [19]. Genetic and biological experiments have detailed the functional properties of ACR4 in developing and mature tissues. The RLK primarily effects epidermal formation in the leaves, sepal margins, and reproductive tissues of the plant [15–16, 18]. Elegant cell biology studies indicate ACR4 influences root development and morphology. Thus, ACR4 is required for columella stem cell differentiation in the root apical meristem and is essential for proper lateral root formation [20–21]. A signaling module has been proposed including a postulated peptide ligand, CLE40, the ACR4 RLK, and the WOX5 transcription factor that engage in a possible feedback mechanism controlling stem cell differentiation similar to CLAVATA signaling in the shoot apical meristem [22–23]. More recently, ACR4 and CLAVATA1 (CLV1) have been shown to be involved in the same pathway to restrict root stemness at the root tip. Importantly, there is now evidence that shows ACR4 and CLV1 can form homo- and heterodimers and regulate root meristem maintenance in response to the CLE4 signaling peptide [24].

The *Arabidopsis* genome encodes four homologs to ACR4, termed *Arabidopsis* CRINKLY4-Related proteins (AtCRRs), which are similar in sequence and architecture to ACR4 [17]. Both CRR1 and CRR2 have been described as kinase-defective due to the absence of the activation loop, a stretch of sequence critical for activity [17, 25]. Recombinant CRR2 kinase domain has been shown to have little to no activity *in vitro* [17] but can be phosphorylated by ACR4 kinase, suggesting a possible intermolecular association. Functional redundancy has been suggested to account for the five members of the ACR4 gene family [17]. Developing *Arabidopsis* roots showed significant enhancement of lateral root densities in *acr4*/*crr* triple mutant backgrounds compared to *acr4* single mutants, suggesting that the CRRs may be able to compensate for loss of ACR4 function in restricting lateral root initiation [21]. Various genetic and cell biology experiments have hinted at multiple players/interactions potentially involved in the ACR4 signaling network. However, the molecular aspect of these interactions with potential membrane-associated or cytosolic protein targets at the cell surface is vaguely understood.

To better understand the role of protein-protein interactions mediated by ACR4 *in vivo*, we have undertaken an *in vitro* study of the interactions between the intracellular domains (ICD) of ACR4 and the CRRs. We have utilized *in vitro* kinase assays to demonstrate that ACR4 can phosphorylate the CRRs and can interact with all four CRRs in the unphosphorylated state. However, phosphorylation dependency is observed for the interaction between ACR4 and CRR3. We also demonstrate through peptide binding assays that a conserved ‘KDSAF’ motif in the helix-C region of CRR3 and CRK1 can bind to the ACR4 kinase domain. For the first time, we also provide evidence for the direct interaction of ACR4 ICD with WOX5. Importantly, ACR4 can phosphorylate WOX5 *in vitro* and mass spectrometry analysis has identified four phosphorylation sites within the WOX5 protein sequence.

## Materials and Methods

### Vector construction

Constructs of SUMO fused ACR4 ICD (sACR4), mutant ACR4 derivatives, and the CRR ICDs (sCRRs) were prepared as described in Meyer et al [26]. The ICD of ACR4 was also cloned as a C-terminal fusion to Maltose Binding Protein (MBP). First, the ACR4 ICD was PCR amplified using the forward primer 5’- GCGGGCGCGGCCTTAAGGTACAGATTGAGGAATTGT-3’ and the reverse primer 5’- CCAACCGCGGCCTCAGAAATTATGATGCAAGAACAAG-3’. The PCR amplified gene was then cloned by standard ligation independent cloning (LIC) procedures into an in-house modified pMAL-C2E vector generated for LIC and containing a 6X His tag at the amino-terminus of MBP. DNA sequencing confirmed correct insertion of the ACR4 ICD gene into the pMAL-C2E vector.

The WOX5 cDNA was synthesized (GenScript) with codon optimization for *E*. *coli* expression and synthesized. The gene was cloned as a C-terminal fusion to MBP (mWOX5) using the forward primers 5’-GCGGGCGCGGCCTTAGGTAGTTTTAGTGTGAAAGGC-3’ and the reverse primer 5’-CCAACCGCGGCCTCATTACAGAAACGACAGACGCAGG-3’. The resulting insert was cloned as describe above, into an in-house modified pMAL-C2E vector generated for LIC but contained a 6X His tag at the carboxy-terminus to aid in purification of full length mWOX5 protein.

### Mutagenesis

To generate the inactive kinase mutants, the catalytic Asp residue of each kinase was mutated to Ala. The vectors encoding the SUMO-fused intracellular domains of the CRRs (sCRRs) were mutagenized using the QuickChange Lightning Multi Site-Directed Mutagenesis Kit (Stratagene) using the corresponding primers:

CRR1, 5’-GAATCATTCATGGCGCTGTGAAGTCTTCGAATG-3’;

CRR2, 5’-CCGATAATTCACAGAGCTGTGAAAACGTCCAAC-3’;

CRR3, 5’-CCACCGATTATTCATAGAGCTATTAAGTCGTCTAAC-3’;

CRK, 5’-CCGGTGATCCACCGTGCCATAAAGTCATCAAAC-3’;

The inactive sACR4–2m vector was prepared as previously described (26). Similarly, mutants of ACR4 with Ala mutations in the LLSLL motif in the N-terminal lobe were made by first creating the mutant KC-LLAAL using the primer 5’-CTCAACCATGCTCATCTTCTTGCCGCTCTTGGATACTGTGAAGAATGG-3’. This mutant was subsequently used as a template to make the KC-AAAAA mutant (all residues in the LLSLL motif replaced with Ala) using the primer 5’-CTCAACCATGCTCATGCTGCTGCCGCTGCTGGATACTGTGAAGAATGG-3’.

### Protein expression and purification

Expression and purification was performed as described by Meyer et al [26]. Proteins were expressed for 5h in Rosetta2 (DE3) pLysS (Novagen) cells at 20°C with 1mM IPTG induction and the proteins purified as described (26). Briefly, pellets from 250 ml cultures were resuspended and lysed in 10 ml Lysis Buffer (50 mM Tris-HCl pH 7.4, 150 mM NaCl, 40 mM imidazole, 0.1% Triton X-100, 1 mM DTT, and 1 mM AEBSF). After centrifugation the supernatant lysate was incubated with Ni-NTA Superflow resin (Qiagen) and bound proteins eluted with Elution Buffer (50 mM Bis-Tris pH 7.2, 50 mM NaCl, 150 mM imidazole, 1 mM DTT) on ice. Protein quantities were determined by the Bradford method [27] and yields typically ranged from 1–5 mg of protein.

To isolate the MBP fused ACR4 kinase (mACR4), the vector was transformed into T7 Express cells (New England Biolabs) and plated on Ampicillin plates. Protein expression and purification was performed as described above, except Ampicillin (100 μg/ml) was used in cultures. Further purification of the IMAC enriched protein was performed by gel filtration on an FPLC system using the 10/300 Superdex G200 column (GE Healthcare) equilibrated with Column Buffer (50 mM Tris-HCl 7.4, 100 mM NaCl, 1 mM DTT). The MBP-fused WOX5 (mWOX5) protein was also expressed and purified as described above.

### Circular Dichroism (CD) Measurements

Far UV CD spectra of sACR4 KC and the mutant, sACR4 KC-AAAAA were recorded with a Jasco-710 spectropolarimeter in a 0.1 cm path length cuvette with excitation wavelengths ranging from 190–260 nm. The protein sample (A_280_ of ~1) was prepared in 10 mM Tris-HCL (pH 7.4) buffer containing 0.1 mM TCEP.

### Peptide Synthesis

Synthetic peptides for a 15 amino-acid segment corresponding to (a) the sequence ^468^DTRSS**KDSAF**TKDNG^482^ in the juxtamembrane domain of ACR4 ICD (b) the sequence ^487^HRRAD**KDSAF**VNELES^502^ in the C-helix of the kinase domain of CRK1 and (c) the sequence ^533^KKFQE**KETAF**DSEIAF^548^ in the C-helix of the kinase domain of CRR3 were synthesized with a biotin tag at the amino terminus (Genscript, USA).

### Pull-down assays

The mACR4 kinase (20 μg per reaction) was used as bait to pull out sCRR kinase domains (30 μg per reaction). Depending on the assay conditions, proteins were autophosphorylated in proteins 200 μl Kinase Buffer (20 mM Bis-Tris pH 7.2, 25 mM NaCl, 5 mM MnCl2, 1 mM DTT) and 100 μM ATP. Proteins were allowed to autophosphorylate for 1 h prior to pull-down experiments. Potential binding partners were added to each reaction and Binding Buffer (50 mM Tris-HCl pH 7.4, 150 mM NaCl, 1 mM DTT, 0.02% NP-40) was added to a final volume of 500 μl. Proteins were incubated on a rotator for 1 h at room temperature. 25 μl bed volume of amylose resin was added to each reaction and incubated an additional 1 h on a rotator at room temperature. Reactions were spun down at 1,000 rpm for 30 sec to pellet the resin. The resin was washed three times with Binding Buffer to remove unbound protein. After the third wash, the supernatant was removed and 100 μl of Laemmli buffer was added to the resin. The resin/protein mixture was boiled at 95°C for 5 min then centrifuged at max speed to pellet resin. 10 μl of supernatant from each reaction was separated by 12% SDS-PAGE and proteins were analyzed by coomassie staining or by western blots with polyclonal anti-MBP (Rockland Immunochemicals, 1:3,000 dilution) or anti-SUMO (Rockland Immunochemicals, 1:2,000 dilution).

The kinase active proteins were used for conditions requiring phosphorylated or unphosphorylated proteins of both interacting partners. The inactive kinase mutants were used for conditions which required one protein of the interaction pair to be unphosphorylated. First, mACR4 protein was autophosphorylated as described above, immobilized on 25 μl of amylose resin, and washed three times with Binding Buffer to remove excess ATP.

mWOX5 protein was used to pull down sACR4 protein similar to the protocol outlined above. Briefly, 20 μg of mWOX5 protein was incubated with 30 μg of sACR4 protein. Depending on the assay conditions, sACR4 was autophosphorylated as described above. mWOX5 and sACR4 were added to a reaction and Binding Buffer was added to a final volume of 500 μl. Proteins were incubated and pulled-down as described above. 10 μl of supernatant from each reaction was separated by 10% SDS-PAGE and proteins were analyzed by coomassie staining or by western blots with polyclonal anti-MBP (Rockland Immunochemicals, 1:3,000 dilution) or anti-SUMO antibody (Rockland Immunochemicals, 1:2000 dilution).

### Gel filtration studies

Purified sACR4 protein was incubated with a sCRR protein at equimolar concentrations to determine possible interactions. 5 μM sACR4 was incubated with 5 μM of binding partner in 500 μl of Column Buffer (50 mM Tris pH 7.4, 100 mM NaCl, 1 mM DTT, and 100 μM MnCl_2_) for 1 h at room temperature. Protein mixtures were then loaded onto a Global 10/300 Superdex G-200 Column (GE Healthcare) coupled to an AKTA FPLC system. Proteins were passed through the column at a flow rate of 0.5ml/min and 1 ml fractions were collected. Proteins in subsequent fractions were analyzed by 12% SDS-PAGE.

### *In vitro* phosphorylation assays

Active sACR4 protein was incubated with the inactive sCRRm proteins. For each reaction 1 μg of sACR4 was incubated with ~1 μg of sCRRm in 20 μl of Kinase Buffer (20 mM Bis-Tris pH 7.2, 25 mM NaCl, 5 mM MnCl_2_, 1 mM DTT, 25 μM ATP) supplemented with 2 μCi of [γ-^32^P] ATP (Perkin Elmer, 6,000 Ci/mmol). Proteins were incubated at room temperature for 1 h. Reactions were then terminated with the addition of 4X SDS sample buffer and boiled. Proteins were separated by 12% SDS-PAGE and radioactive bands were analyzed by phosphorimaging.

A modified *in vitro* phosphorylation assay was used for sACR4 and mWOX5 since they migrate similarly on an SDS-PAGE gel. Phosphorylation proceeded in a 50 μl reaction containing 2 μg of sACR4 and 30 μg of mWOX5 (or 30 μg of MBP as a control) in Kinase Buffer as described above. Following incubation, mWOX5 protein was pulled-down using amylose resin. The phosphorylation reactions were brought up to 500 μl with Wash Buffer (50 mM Tris-HCl pH 7.4, 150 mM NaCl, 1 mM DTT, and 0.5% NP-40). 25 μl bed volume of amylose resin was added to each reaction and incubated an additional 1 h on a rotator at room temperature. Reactions were spun down to pellet the resin and the supernatant was removed. The resin was washed five times with 500 μl of Wash Buffer to remove unbound protein. After washing, the supernatant was removed and 100 μl of Laemmli buffer was added to the resin. The resin/protein mixture was boiled at 95°C for 5 min then centrifuged to pellet resin. Proteins from each reaction were separated by 10% SDS-PAGE and analyzed by phosphorimaging.

### Peptide overlay assays

These were performed as described previously (26). Briefly, the target proteins (sACR4 KC, sACR4 KC-AAAAA and SUMO) were blotted on to a nitrocellulose membrane after separation by 12% SDS-PAGE. After staining with a 0.1% Ponceau S solution, bands were then cut from the blot and destained. After washing and blocking steps, individual blotted bands were then added to microfuge tubes containing 1 ml of the appropriate peptide (synthesized with an N-terminal biotin tag) at a final concentration of 100 μM in Tris-HCl buffer at pH 8.0. Blots were incubated on a rotating platform for 2 h at room temperature, followed by two washes with TBS at 5 min intervals. Peptides were also incubated with SUMO protein as a control. Peptide binding was detected by incubation with streptavidin conjugated Alkaline Phosphatase (AP) (Pierce) and AP Substrate kit (Bio-Rad) as described previously [26].

### Peptide binding assays

The binding of ‘KDSAF’ peptides to sACR4 KC, sKC-AAAAA and SUMO (control) protein was performed as described previously [26]. Briefly, the proteins were first immobilized in a 96 well nickel coated plate (XpressBio) by incubating 100 μl of ~1 μM protein sample in TBS + 0.1% Tween20 for 1 h at room temperature. After a few washing steps, wells were subsequently incubated with 50 μl of the appropriate peptide solution (200 μM) on an orbital shaker for 2 h at room temperature. Excess peptide solution in the wells was then discarded, washed with TBS to remove unbound peptides and incubated with 50 μl of a streptavidin-AP solution (1:500) for 1 h at room temperature. Colorimetric detection of peptide binding was initiated by adding 100 μl of a pNPP solution (100 mM ethanolamine pH 9.8, 10 mM pNPP) to each well. The reactions were allowed to incubate 5 min with occasional manual shaking. Reactions were terminated with the addition of 50 μl of 2N NaOH to each well. 100 μl of each reaction was diluted with 400 μl of water and absorbance at 405 nm measured. Each peptide binding experiment was performed in triplicate and data represented as the mean ± the S.E. Data were corrected for dilution factor and subtracting the absorbance of the control (SUMO) well from the experimental samples.

### Identification of WOX5 phosphorylation sites by mass Spectrometry

This was performed as described by Meyer et al. [19]. Briefly, tryptic peptides were generated from the reduced-alkylated protein using Sequencing Grade Modified Trypsin (Promega). The resulting peptides were desalted on a SOURCE 15 RPC column (GE Healthcare) and eluted in 90% acetonitrile, 0.1% TFA. The eluted peptides were then dried down in a vacuum centrifuge. Phosphopeptides were enriched using the PHOS-Select Ga^3+^ Silica Spin Column kit (Supelco) according to the kit instructions. The complex peptide mixtures were analyzed using high-resolution nano-LC-MS on a hybrid mass spectrometer consisting of a linear quadrupole ion-trap and an Orbitrap (LTQ-Orbitrap XL, Thermo Fisher Scientific). Chromatographic separations were performed using a NanoLC-1D Plus (Eksigent) for gradient delivery and a cHiPLC-nanoflex (Eksigent) containing a 15 cm x 75 μm C18 column (ChromXP C18-CL, 3 μm, 120 Å, Eksigent). The liquid chromatograph was interfaced to the mass spectrometer with a nanospray source (PicoView PV550; New Objective). The survey scans (*m/z* 350–2000) (MS1) were acquired at high resolution (60,000 at *m/z* = 400) in the Orbitrap and the MS/MS spectra (MS2) were acquired in the linear ion trap at low resolution, both in profile mode. The MS1 scan was followed by one MS2 event with collision activation in the ion trap (parent threshold = 10000; isolation width = 4.0 Da; normalized collision energy = 30%; activation Q = 0.250; activation time = 30 ms). The following ion source parameters were used: capillary temperature 200°C, source voltage 3.3 kV, source current 100 μA, capillary voltage 34 V, and the tube lens at 125 V.

### Data Analysis

The MS2 spectra were analyzed by searching against the WOX5 sequence and expert manual interpretation. The exact masses of the phosphopeptide and fragmentation ions were calculated using the MS-Product utility within Protein Prospector (http://prospector.ucsf.edu). For database searches, the LC-MS files were processed using MASCOT Distiller (Matrix Science, version 2.3.0.0) with the settings previously described (19). The resulting MS2 centroided files were used for database searching with MASCOT, version 2.1.6, against a custom, in-house database containing 266 disparate proteins including the WOX5 sequence using the following parameters: enzyme, trypsin; MS tolerance = 15 ppm, MS/MS tolerance = 0.8 Da with a fixed carbamidomethylation of Cys residues and the following variable modifications: oxidation (Met) and phosphorylation (Ser, Thr, and Tyr); Maximum Missed Cleavages = 9; and 1+, 2+ and 3+ charge states. For analysis of the tandem spectra from spectral acquisitions in the Orbitrap (MS2), the raw files were processed using MASCOT Distiller (Matrix Science, Oxford, UK) with the following settings: 1) as described previously [19]. The searched data was analyzed in Scaffold_4.4.1.1 (Proteome Software). A 0.1% false discovery rate (FDR) was calculated using protein probabilities estimated from the results of the Protein Prophet algorithm with threshold settings of 95% for peptides and proteins and a minimum of two peptides per protein.

### Hydrogen/Deuterium Exchange Mass Spectrometry

Differential, solution phase HDX experiments were performed to detect the conformational change of sACR4 induced by the addition of sCRK1. Each exchange reaction was initiated by incubating 4 μl of protein complex (with or without sCRK1) with 16 μl of D_2_O protein buffer (50 mM Bis Tris pH 7.2, 25 mM NaCl, 0.1 mM MnCl_2_, 1 mM DTT) for a predetermined time (10, 30, 60, 120, 900, and 3600 s) at 4°C. The exchange reaction was quenched by mixing with 30 μl of 3 M urea, 1% TFA at 1°C. The mixture was passed across an in-house packed pepsin column (2 mm X 2 cm) at 200 μl/min and digested peptides were captured onto a 2mm X 1cm C_8_ trap column (Agilent) and desalted (total time for digestion and desalting was 3 min). Peptides were then separated across a 2.1 mm X 5 cm C_18_ column (1.9μ Hypersil Gold, Thermo Scientific) with linear gradient of 4%–40% CH_3_CN, 0.1% formic acid, over 5 min. Protein digestion and peptide separation were performed within an ice water bath to reduce D/H back exchange. Mass spectrometric analyses were carried out with capillary temperature at 225°C, and data were acquired with a measured resolving power of 100,000 at m/z 400. Three replicates were performed for each on-exchange time point.

### Peptide Identification and HDX Data Processing

MS/MS experiments were performed with a LTQ Orbitrap mass spectrometer (ThermoFisher). Product ion spectra were acquired in a data-dependent mode and the six most abundant ions were selected for the product ion analysis. The MS/MS *.raw data files were converted to *.mgf files and then submitted to Mascot (Matrix Science, London, UK) for peptide identification. Peptides included in the peptide set used for HDX had a MASCOT score of 20 or greater. The MS/MS MASCOT search was also performed against a decoy (reverse) sequence and ambiguous identifications were ruled out. The MS/MS spectra of all of the peptide ions from the MASCOT search were further manually inspected, and only those verifiable are used in the coverage. The intensity weighted average m/z value (centroid) of each peptide isotopic envelope was calculated with HD Desktop [28]. The deuterium level was calculated as described previously [29]. Deuterium level (%) = {[m(P)—m(N)]/[m(F)—m(N)]} X 100%, where m(P), m(N) and m(F) are the centroid value of partly deuterated peptide, nondeuterated peptide, and fully deuterated peptide, respectively. The correction of accounting for the known 80% deuterium content of the on-exchange buffer was made. No back exchange correction was made and all values are therefore reported as relative.

## Results

### *In vitro* interactions between the ACR4 and CRR ICDs

**Fig 1:**
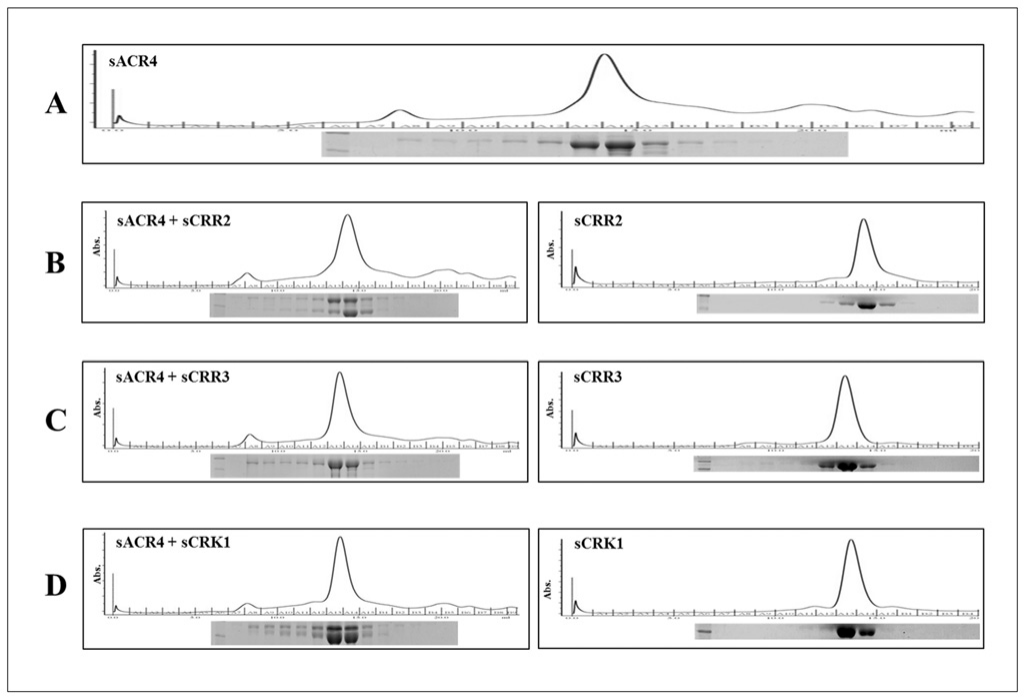
Gel filtration analysis of ICD interactions. Protein-protein interactions between the ICDs of ACR4 and the CRR binding partners were assessed by gel filtration on a Superdex G-200 column. (A) ACR4 elution profile demonstrating the protein primarily elutes as a monomer, but has the propensity to form higher order oligomers. (B-D) the elution profiles of the CRRs incubated alone (*right panels*) or with ACR4 (*left panels*). Insets depict 12% SDS-PAGE gels of eluted protein fractions.

We examined potential interactions between the ICDs of ACR4 and the CRRs by gel filtration chromatography (Fig. 1) using recombinantly expressed SUMO-tagged proteins. Pairs of unphosphorylated proteins were incubated together at equimolar concentrations (5 μM each) and subjected to gel filtration. It is evident by this analysis that purified sACR4 protein is predominantly monomeric, although there is evidence of higher order oligomers (Fig. 1A and inset). This is in agreement with our previous gel filtration studies of the NusA fused ACR4 ICD [19] and indicates that the solubility tag does not influence oligomerization. When sACR4 was incubated with sCRR2 or sCRK1, the gel filtration profile clearly indicated interactions between these proteins. Thus, the elution profiles showed the formation of oligomeric complexes containing sACR4/sCRR2 (Fig. 1B, left panel) and sACR4/sCRK1 (Fig. 1D, left panel) when analyzed by SDS-PAGE (Fig. 1B and D, *left panels inset*). Importantly, neither sCRR2 (Fig. 1B, right panel) nor sCRK1 (Fig. 1D, right panel) by themselves showed any evidence of self-association in the absence of phosphorylation at the concentrations examined during gel filtration. These results suggest that ICDs of CRK1 and CRR2 can interact with the ICD of ACR4 in the absence of phosphorylation. No oligomeric complexes were observed with the sACR4/sCRR3 samples. The interaction between sACR4 and sCRR1 could not be studied by gel filtration owing to the propensity of sCRR1 to aggregate under these conditions.

### Effects of phosphorylation on heteromeric interactions

**Fig 2:**
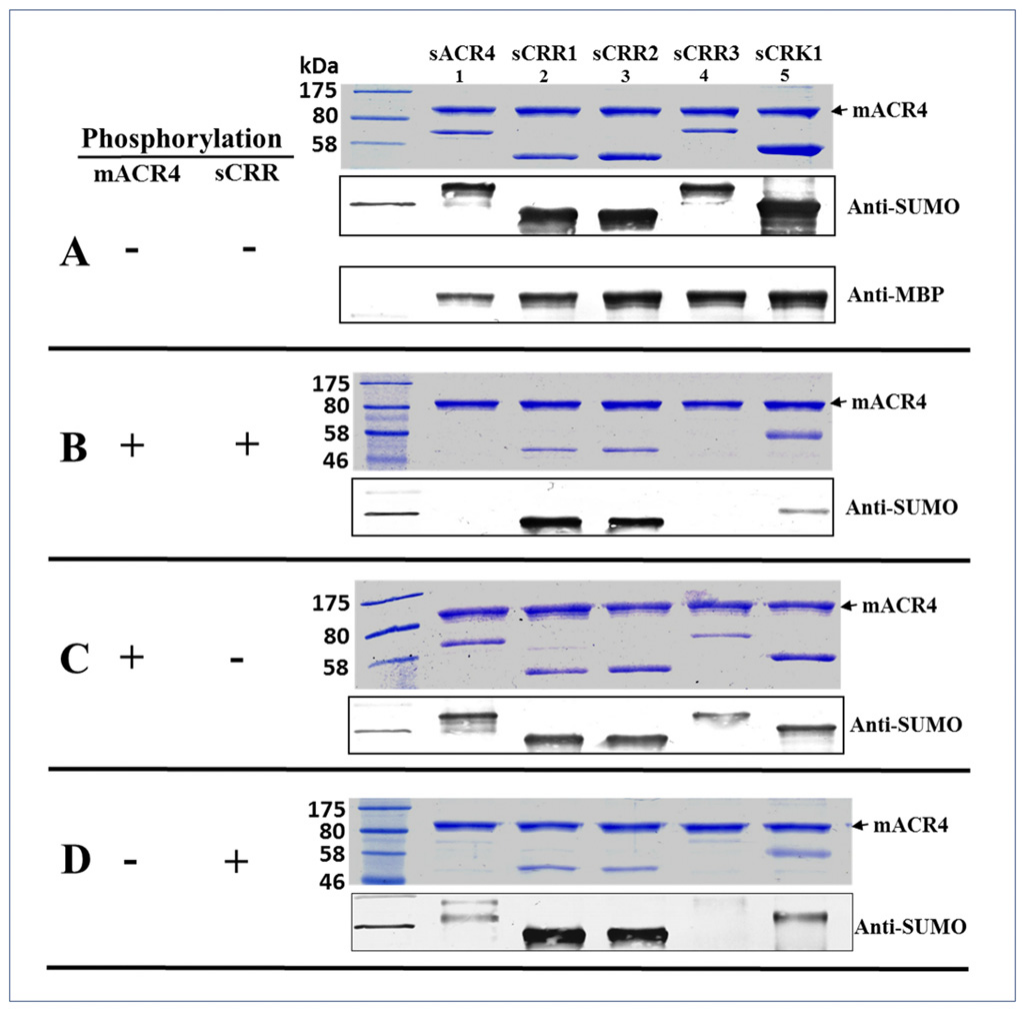
Effect of autophosphorylation on ICD interactions. Pull-down assays were performed to determine the effects of phosphorylation on interactions between the ICDs of ACR4 and the CRRs. The autophosphorylation status of each protein was varied for individual experiments, (+) denotes autophosphorylated protein, (-) denotes unphosphorylated protein. In (A), mACR4 (-) and sCRRs (-), (B) mACR4 (+) and sCRRs (+), (C) mACR4 (+) and sCRRs (-), (D) mACR4 (-) and sCRRs (+). In A-D, proteins were separated by 12% SDS-PAGE (*top panels*) and the presence of interacting proteins was determined by anti-SUMO western blot (*lower panels*). In the bottom panel of A, anti-MBP western blot demonstrates the presence of mACR4 in each reaction.

The kinase domains of many plant receptors are known to undergo phosphorylation during receptor activation and oligomerization [30–32]. To determine if the associations between the ICD of ACR4 and the ICDs of the CRRs were influenced by phosphorylation, *in vitro* pull-down experiments were performed with various combinations of autophosphorylated kinases (Fig. 2A-D). The MBP-fused ICD of ACR4 (mACR4) protein (~0.4 μM) was used as the bait and the sCRRs proteins (~1 μM) were used as the prey. Therefore, the target proteins were in ~ 2.5 molar excess over the mACR4 bait protein. In the naïve, unphosphorylated state, mACR4 was able to pull out all of the naïve sCRR proteins (Fig. 2A). This suggests that these kinases possess the intrinsic propensity to hetero-oligomerize in the unphosphorylated state. It is worth noting that this experiment was able to demonstrate an interaction between the naïve mACR4 and sCRR3 when the concentration of sCRR3 was in excess of sACR4, suggesting that concentration effects may indeed drive hetero-association of these two proteins not seen in our gel filtration studies that were performed at equimolar protein concentrations. Additionally, the SUMO tagged ACR4 (sACR4) intracellular domain was pulled out by mACR4 confirming previous sedimentation velocity experiments using the NusA fused ACR4 ICD that indicated kinase domain oligomerization in the absence of phosphorylation [19]. Control reactions showed these interactions were specific towards the ICDs and were not due to interactions with the SUMO and MBP solubility tags or nonspecific binding of fusion proteins to the amylose resin (data not shown). The presence of mACR4 in each reaction was confirmed with western blots using polyclonal, anti-MBP antibody (Fig. 2A, *lowest panel*) and the sCRRs were confirmed by western blots probed with a polyclonal, anti-SUMO antibody (Fig. 2A-D, *lower panels*).

Interestingly, autophosphorylation of the ICDs had striking effects on ACR4 interactions with the CRR homologs. Loss of interaction occurs between mACR4/sACR4 (Fig. 2B, *lane 1*). The fact that phosphorylated mACR4 does not interact with phosphorylated sACR4 supports our previous observation that ACR4 monomerizes upon autophosphorylation [19]. The interaction between phosphorylated mACR4 and phosphorylated sCRK1 is attenuated but not completely abolished. (Fig. 2B, *lane 5*). Intriguingly, the interaction between sACR4 and sCRR3 is completely abolished, demonstrating that autophosphorylation of the ICDs forces dissociation of these two proteins (Fig. 2B, *lane 4*). The dissociation may be due to conformational changes that accompany autophosphorylation [19] and/or electrostatic repulsion due to increases in net negative charge from autophosphorylation.

The interactions observed between the mACR4/sACR4 and mACR4/sCRR3 showed decreased binding ability depending on whether the bait protein (mACR4) or the target protein (sACR4, sCRR3) was in the phosphorylated state (Fig. 2C and D) as compared to the two extremes of where the participating proteins are either in the unphosphorylated state (Fig. 2A) or phosphorylated state (Fig. 2B). These results indicate that autophosphorylation of both partners is required to disrupt the intracellular contact between ACR4/ACR4 and ACR4/CRR3 kinase domains. The fact that mACR4 is able to strongly interact with sCRR1 and sCRR2 regardless of phosphorylation state (Fig. 2A-D), raises the possibility that ACR4 may be able to signal through the use of dead kinase domains [33–34].

### ACR4 can phosphorylate the CRR homologs

**Fig 3:**
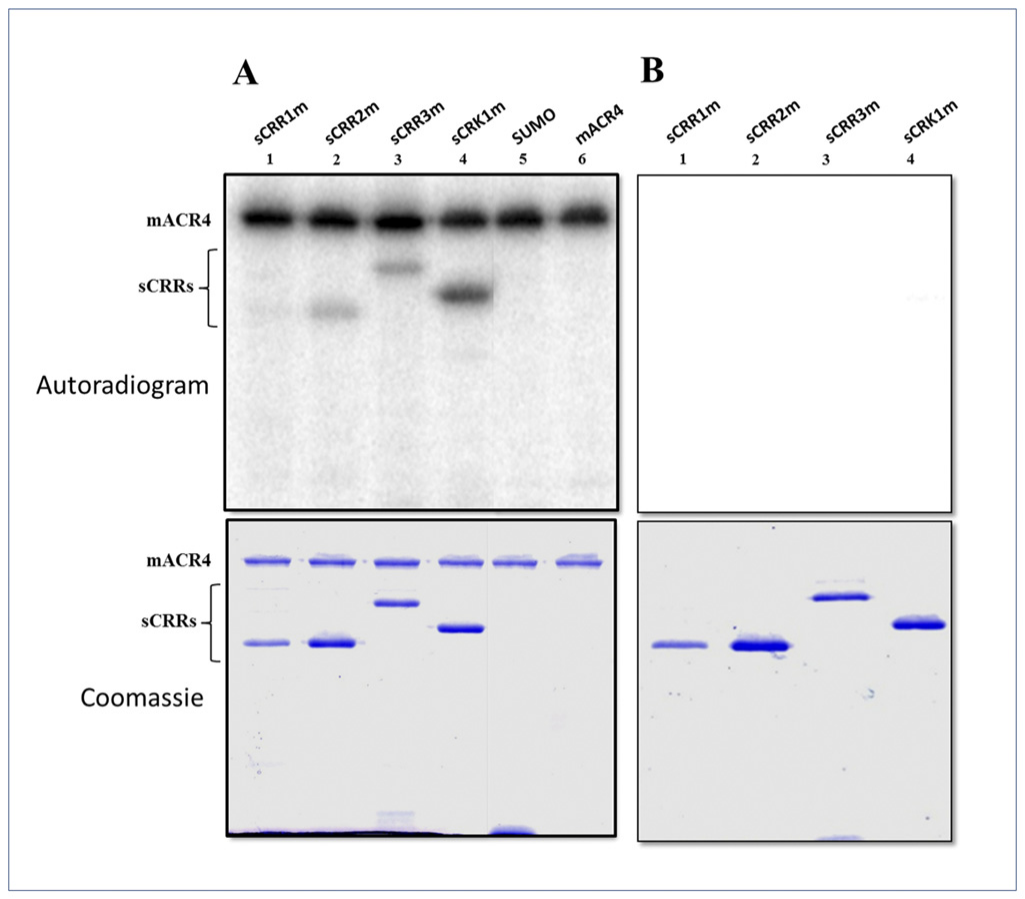
ACR4 can phosphorylate the ICDs of the CRRs. Active mACR4 was incubated with the inactive sCRRs in an *in vitro* kinase assay. (A) *Upper panel* shows an autoradiogram demonstrating ACR4 can phosphorylate the ICDs of the inactive CRRs. ACR4 *cannot* phosphorylate the SUMO tag. *Lower panel* shows the corresponding coomassie stained gel. (B) *Upper panel* depicts an autoradiogram showing the inactivity of the sCRR mutants in which their respective catalytic Asp residues have been mutated to Ala. *Lower panel* is the corresponding coomassie stained gel showing the presence of each protein.

Receptor activation through homo- or heterodimerization is accompanied by intra- and intermolecular phosphorylation reactions, recruitment of proteins to the intracellular domain of the receptor and subsequent activation of signaling events inside the cell [1, 10, 14]. Previous studies have proposed that ACR4 can phosphorylate CRR2 in an *in vitro* phosphorylation assay [17]. We further assessed the ability of ACR4 to phosphorylate the CRRs by incubating the active mACR4 kinase with the kinase dead, sCRR mutants (sCRRms) (Fig. 3A). Since CRR3, and CRK1 have robust kinase activity, we mutated the catalytic Asp [35] of each kinase to Ala in order to inactivate each enzyme. Although CRR1 and CRR2 have intrinsically very weak kinase activity attributable to the absence of the activation loop in each protein (data not shown) [17], to completely eliminate autophosphorylation activity, we also mutated the catalytic Asp in both CRR1 and CRR2 to Ala. Inactivity of each mutant was confirmed by an *in vitro* autophosphorylation assay (Fig. 3B). The ACR4 kinase can in fact phosphorylate the intracellular kinase domains of all four CRR homologs *in vitro*. Amongst the CRRs, the weakest phosphorylation activity was seen against sCRR1m (Fig. 3A, *lane 1*) and relatively stronger activity was seen against the sCRK1m ICD (Fig. 3A, *lane 4*). Thus, these *in vitro* experiments suggest that ACR4 may also have the potential to phosphorylate the CRRs *in vivo*. No phosphorylation of the yeast SUMO protein was observed when incubated with sACR4 indicating that the phosphorylation of the SUMO-tagged CRRs was specific to the intracellular domain and not to the fusion tag (Fig. 3A, *lane 5*).

### Mapping ACR4/CRK1 interaction sites by H/D exchange

To assess the molecular basis of the ACR4-CRR interactions, we used hydrogen-deuterium exchange coupled to mass spectrometry in order to map potential intermolecular interactions sites within the ACR4 ICD. Hydrogen-deuterium exchange (HDX) is an effective tool to map in solution protein-ligand interaction sites and determining conformational dynamics of proteins [36–37]. This technique makes advantageous use of backbone amide hydrogens and their availability for isotopic labeling with deuterium from the solvent. Amide hydrogens undergoing intra- or intermolecular hydrogen bonding have reduced exchange rates when compared to amide hydrogens exposed to the solvent. Therefore, hydrogen exchanged with deuterium can be monitored by mass spectrometry to delineate specific regions within a protein sequence that are solvent accessible. Binding interfaces within a protein complex can be determined by comparing HDX rates of the bound and unbound state [38–40]. Consequently, mapping of isotopically labeled peptides along a given protein sequence provides a general idea of potential protein-protein interactions sites.

**Fig 4:**
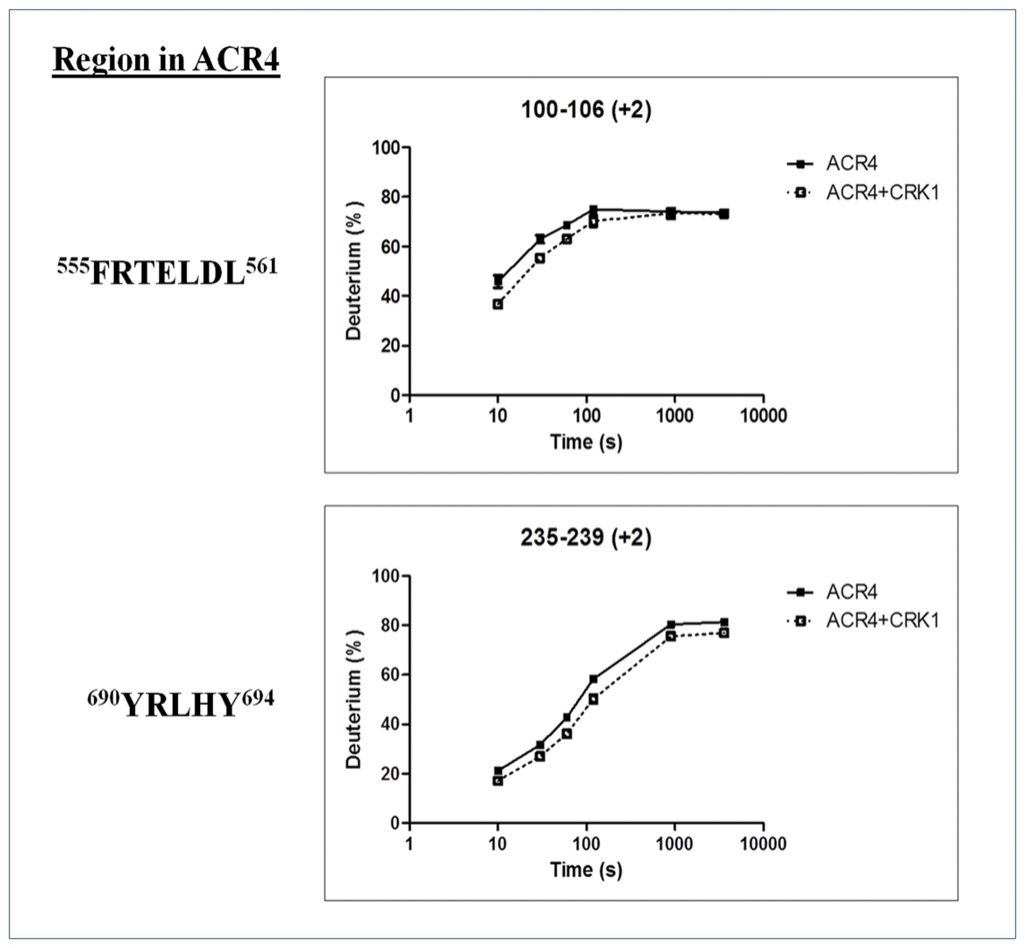
HDX-MS analysis of ACR4/CRK1. Time course of deuterium uptake for two ACR4 peptides, FRTELDL and YRLHY, that demonstrated significant hydrogen-deuterium exchange rates between the free ACR4 (closed squares) and the ACR4/CRK1 bound (open squares) states. Data represent the mean (n = 3).

On the basis of the gel filtration, pull-down, and phosphorylation studies, we determined that CRK1 was a good candidate for mapping potential interactions sites between the unphosphorylated ACR4 and CRK1. The experiment was initially performed with the unbound, *apo*-sACR4 protein to determine individual HDX profiles for all peptides produced from the in-line pepsin digest of the sample. Overall, 100 peptides with overlapping sequence corresponding to the ACR4 ICD were identified in the mass spectrometry analysis of peptic fragments generated from the *apo*-sACR4 enzyme. Peptide sequence coverage was ~98% of the ACR4 intracellular domain. This data was then compared to peptide HDX profiles generated from the *holo*-sACR4 protein bound to sCRK1 after prior incubation at ratios of 1:1 or 1:3 (see Materials and Methods). Evaluation of the mass spectrometry data revealed two sites of protection within the ACR4 ICD. One site, corresponding to the peptic fragment FRTELDL, is a part of helix-C in the N-terminal lobe of the kinase domain. This region displayed a significant average decrease in percent deuteration of 5%. The second peptide, YRLHY, occurs in the C-lobe of the kinase domain, just downstream of the C-terminal hinge of the activation loop, and showed a 6% average decrease in deuterium uptake compared to *apo*-ACR4 (Fig. 4). The decrease in deuterium uptake is indicative of a significant involvement of these two regions in interfacial contacts between ACR4 and CRK1. Our experiments did not reveal any differences in HD exchange for peptides corresponding to the JMD or CTD of ACR4. However, further studies are needed to ascertain the exact role of these subdomains in potential protein-protein contacts.

### ‘KDSAF’ motif binds ACR4 kinase domain

**Fig 5:**
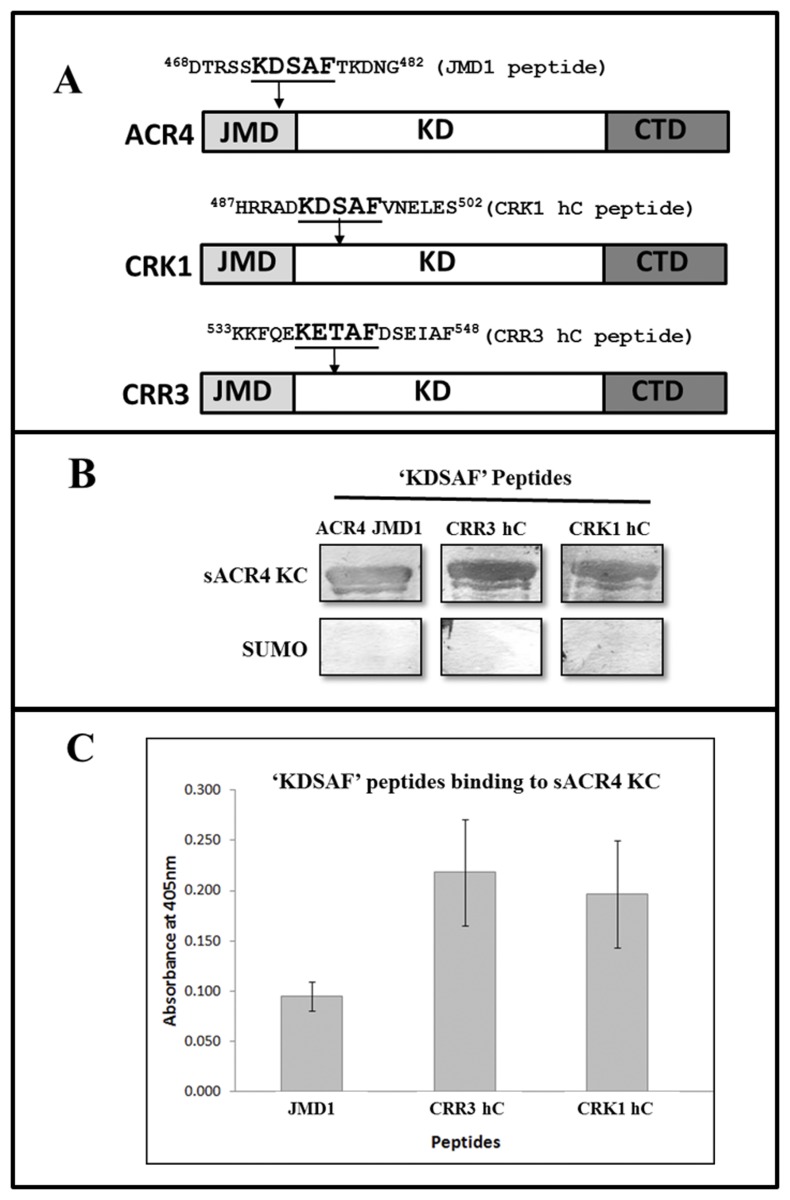
‘KDSAF’ peptides bind to the ACR4 kinase. **(A)** Schematics of the ACR4, CRK1 and CRR3 amino acid sequence. The location of the “KDSAF” motif sequence in the domain architecture of the respective proteins is indicated by an arrow. (B) The **‘**KDSAF’ motif containing peptides were incubated with the sACR4 KC protein to demonstrate specificity of binding towards the ACR4 kinase. A peptide overlay assay showing that the unphosphorylated JMD1 [26], the CRR3 hC and CRK1 hC peptides can bind to sACR4 KC (*upper panel*). Control blots show no specificity towards the SUMO solubility tag (*lower panel*). (C) Peptide binding assay demonstrating the ‘KDSAF’ containing peptides can bind to the natively folded sACR4 KC protein immobilized on a Ni^2+^ coated plate. Each peptide binding experiment was performed in triplicate and data represented as the mean ± the S.E. Data were corrected for dilution factor and subtracting the absorbance of the control (SUMO) from the experimental samples.

In a recent paper [26], we showed that a synthetic peptide JMD1, ^468^DTRSSKDSAFTKDNG^482^, derived from the juxtamembrane domain (JMD) of ACR4-ICD could bind to the kinase domain *in trans*, perhaps via the LLSLL sequence motif, and postulated a potential intramolecular interaction between the juxtamembrane domain and the kinase domain in the ICD. Interestingly, a close scrutiny of the amino acid sequences of ACR4, CRR3 and CRK1 revealed a highly conserved ‘KD/ESAF’ motif (Fig. 5A). The inactive members of the AtCRR family, CRR1 and CRR2 ICDs, do not contain this motif, suggesting that the ‘KD/ESAF’ region may serve a critical role in the kinase-active members of the ACR4 family. However, whereas the KDSAF sequence is found in the JMD of ACR4, in CRR3 and CRK1 it occurs in the kinase domain towards the N-terminal portion of the presumed helix-C region (Fig. 5A). The identification of conserved KDSAF motifs in ACR4, CRR3 and CRK1 ICDs raised the intriguing possibility that the association among these proteins could be mediated, at least in part, via this motif.

To test this hypothesis, we synthesized peptides corresponding to the helix-C regions of CRR3 and CRK1 (Fig. 5A, termed hC peptides), and the JMD1 peptide which contains the ‘KDSAF’ motif in ACR4, for use in peptide overlay assays (Fig. 5B). As described in Materials and Methods, peptide binding was assessed by colorimetric detection using streptavidin conjugated Alkaline Phosphatase. Our results unequivocally show direct binding of the ‘KDSAF’ containing peptides to the sACR4 KC, a protein lacking the JMD (Fig. 5B, *upper panel*). Importantly, the peptides do not bind to the SUMO protein used as a control (Fig. 5B, *lower panel*). The presence of sACR4 KC or SUMO protein was verified by independent western blots using anti-SUMO antibody (data not shown).

In a parallel experiment we determined peptide binding to sACR4 KC that was immobilized on Ni^2+^ coated plates through the N-terminal histidine tag to ensure that the ‘KDSAF’ peptides binding to sACR4 KC was not an artifact caused by nonspecific binding to a misfolded protein. The data in Fig. 5C, demonstrates that all three ‘KDSAF’ motif containing peptides are able to bind to the sACR4 KC protein to different extents. The CRR3 and CRK1 hC peptides showed the highest binding affinity to sACR4 KC and had similar binding capacity under our assay conditions. The differences in binding affinities seen with these peptides suggest that variations in the flanking residues of the core ‘KDSAF’ motif may influence binding affinity. These peptide binding results provide strong support for the involvement of the ‘KDSAF’ region in intermolecular contacts between ACR4 and CRR3 or CRK1 family members.

### ‘KDSAF’ motif peptides show reduced binding to ACR4 kinase domain containing mutations in the LLSLL region

**Fig 6:**
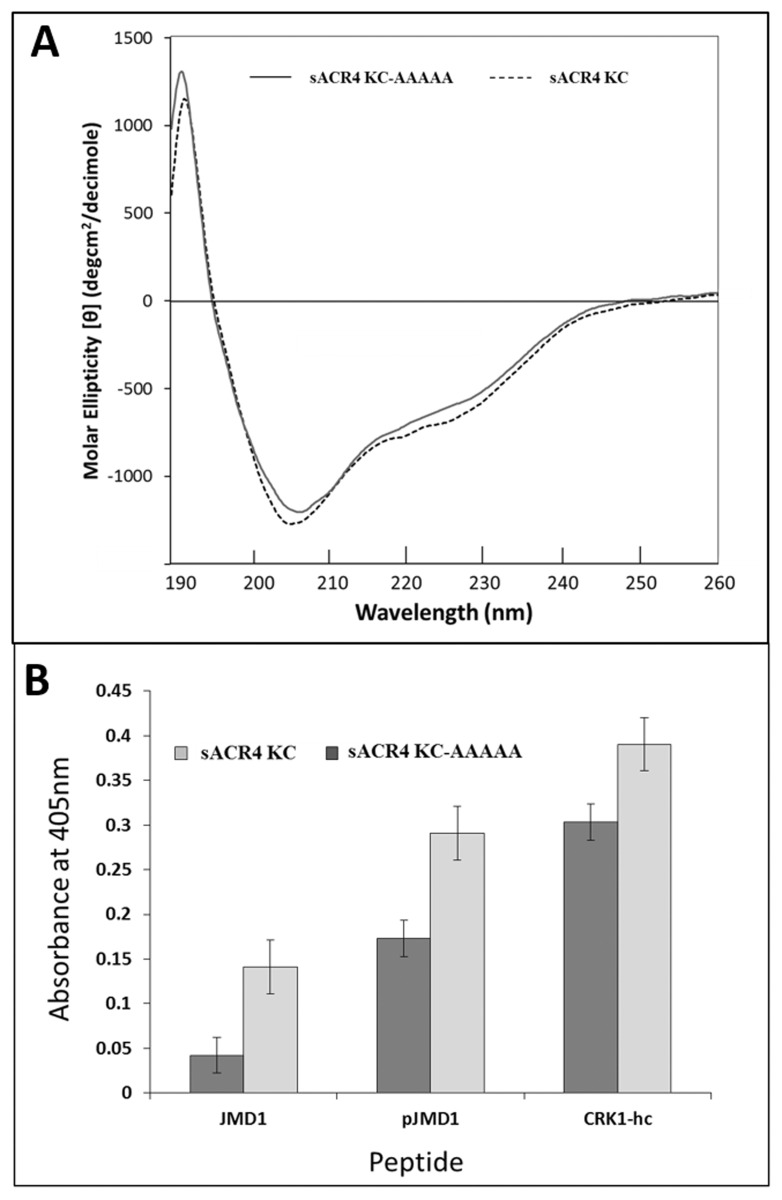
Binding of ‘KDSAF’ peptides to sACR4 KC and sACR4 KC-AAAAA. **(A)** Conformational analysis of sACR4 KC 9 (dashed line) and sACR4 KC-AAAAA (solid line) indicating that mutations did not cause protein misfolding. Far UV CD spectra in 10 mM Tris (pH 7.4) and 0.1 mM TCEP with an A_280_ value of ~1.0. (B) Peptide binding assay demonstrating the binding efficiency of ‘KDSAF’ containing peptides to the sACR4 KC protein and sACR4 KC-AAAAA immobilized on a Ni^2+^ coated plate. The sACR4 KC-AAAAA mutant shows reduced binding affinities for ‘KDSAF’ containing peptides when compared to wild-type sACR4. Each peptide binding experiment was performed in triplicate and data represented as the mean ± the S.E. Data were corrected for dilution factor and subtracting the absorbance of the control (SUMO) from the experimental samples.

Previously we demonstrated that the preferred binding site of the JMD1 peptide in ACR4 is most likely in the N-terminal lobe of the kinase domain, potentially mediated by the LLSLL sequence [26]. To further validate this observation, we first expressed and purified a SUMO-tagged mutant of the ACR4 kinase domain in which the LLSLL sequence was replaced with Ala (s-KC-AAAAA). The CD spectrum of the mutant protein (Fig. 6A) attests to its proper folding. The mutant protein was then immobilized on a Ni^2+^ coated plate and the binding of the ‘KDSAF’ peptides JMD1, pJMD1 (JMD1 with phosphorylated Ser475) and CRK1-hc measured as described above (Fig. 6B). The significant decrease in binding ability to the mutant sKC-AAAA compared with the control KC provides strong support for the involvement of LLSLL region in intermolecular interactions between the ACR4 ICD and the kinase active members of the AtCRR family.

### ACR4 directly interacts with Transcription Factor WOX5

Previous reports have suggested a signaling module involving ACR4 and the WOX5 transcription factor. WOX5 expression dynamics are proposed to be influenced by ACR4 acting through the perception of a postulated CLE40 peptide ligand. ACR4 is primarily expressed in the D1-D3 cell layers in the root tip, whereas WOX5 is solely expressed in quiescent center (QC) [21–22, 41]. Overexpression of the CLE40 gene or application of exogenous CLE40 peptide expands the ACR4 expression domain to include the QC [21–22]. We therefore hypothesized that the ACR4 intracellular domain may have the capability to directly interact with WOX5 and phosphorylate it, thus affecting WOX5 function inside the cell.

**Fig 7:**
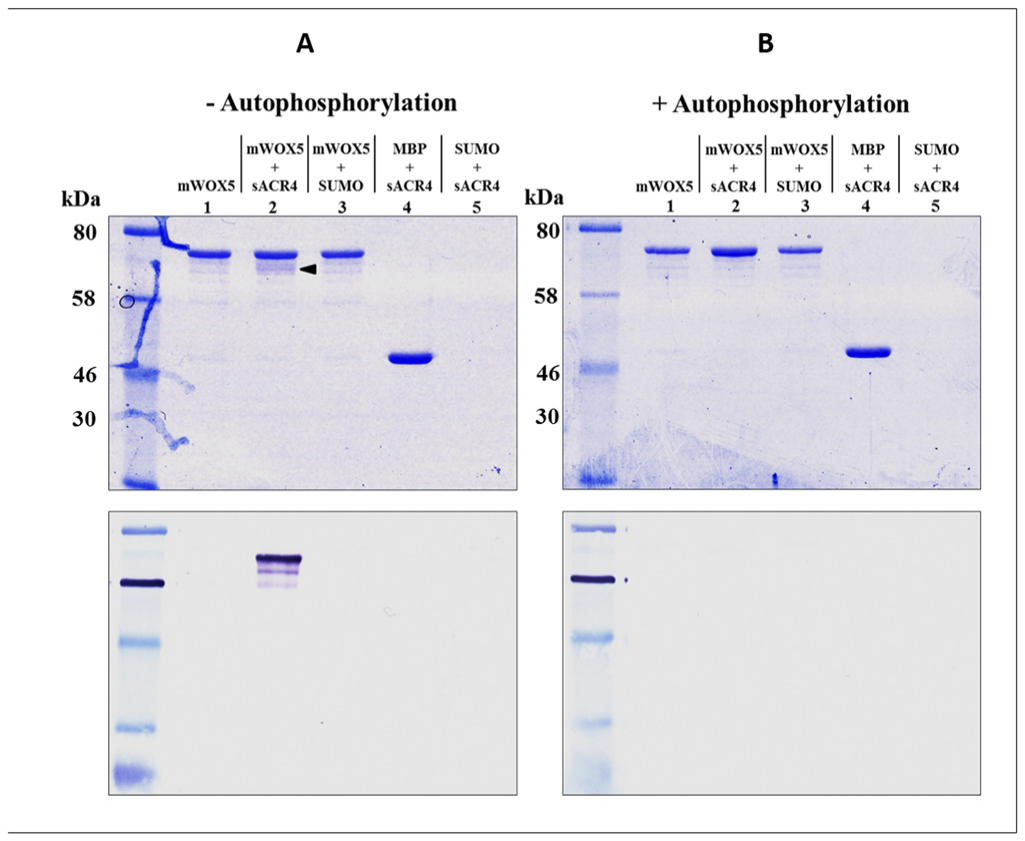
ACR4 ICD interaction with WOX5. The interaction between sACR4 and mWOX5 was determined by an *in vitro* pull-down assay. mWOX5 was incubated in the presence of unphosphorylated or phosphorylated sACR4 in parallel experiments. In (A), mWOX5 can specifically bind and pull-down naïve sACR4 (*lane 2*, *arrow*). An anti-SUMO western blot confirmed sACR4 binding (*lower panel*). In controls, no binding is observed to SUMO alone (*lane 3*) and MBP does not bind to sACR4 *(lane 4)*. In (B) mWOX5 *cannot* interact with autophosphorylated sACR4. In A and B, 12% SDS-PAGE separation of interacting proteins (*top panels*) and anti-SUMO western blots of proteins from each reaction (*lower panels*).

To examine the *in vitro* association of ACR4 with WOX5, we first incubated the purified sACR4 and mWOX5 proteins together and used amylose resin to pull-down the mWOX5 protein. Additionally, in a parallel experiment, we determined the effect of autophosphorylation of the ACR4 ICD on interaction with WOX5. Our results demonstrate that naïve ACR4 is indeed pulled-down by mWOX5 protein (Fig. 7A, *upper panel*, *lane 2*, *arrow*) but this interaction is abolished with phosphorylated ACR4 protein (Fig. 7B, *upper panel*, *lane 2)* indicating that autophosphorylation is not a requirement for association. To unequivocally show that mWOX5 was pulling out sACR4 (Fig. 7A, *upper panel*, *lane 2*, *arrow*), we performed a separate western blot using anti-SUMO antibody (Fig. 7A, *lower panel*, *lane 2*). In addition, control reactions using either the SUMO or MBP solubility tags demonstrate the observed interaction was specifically between the ACR4 ICD and WOX5 (Fig. 7A and B, *lanes 3–5*).

### Mapping of *in vitro* WOX5 phosphorylation sites

**Fig 8:**
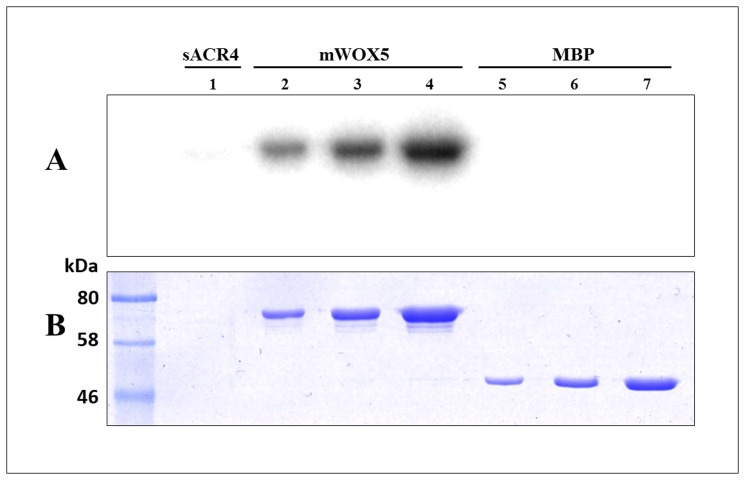
ACR4 can phosphorylate WOX. To demonstrate ACR4 phosphorylation of WOX5, sACR4 was incubated with mWOX5 in an *in vitro* kinase assay. **(A)** An autoradiogram demonstrating phosphorylation of WOX5. The absence of sACR4 (10 μl load) is shown in *lane 1*. Phosphorylated mWOX5 (*lanes 2–4*) was loaded at increasing amounts, 2, 5, and 10 μl of reaction per lane, respectively. A control reaction showing that the MBP tag *cannot* be phosphorylated by ACR4 (*lanes 5–7*) was loaded at increasing amounts, 2, 5, and 10 μl of reaction per lane, respectively. **(B)** The corresponding coomassie stained gel.

The *in vivo* expression patterns of WOX5 and ACR4 have been demonstrated to overlap in the *Arabidopsis* root tip [21–22]. In addition, our pull-down studies indicate that the ICD of ACR4 can interact with WOX5. We therefore considered the possibility that the ACR4 kinase domain could phosphorylate WOX5, thus affecting WOX5 function in the root. Transcription factors can be regulated through reversible phosphorylation, therefore regulating their function inside the cell. For example, STAT transcription factors can be directly phosphorylated by the EGF receptor kinase domain to modulate their DNA binding activity [42–43]. Thus, we incubated sACR4 with mWOX5 in an *in vitro* kinase assay in the presence of [γ-^32^P] ATP. We clearly demonstrate that WOX5 is phosphorylated by sACR4, *in vitro* (Fig. 8, *lanes 2–4*). Since sACR4 and mWOX5 have similar migration patterns on an SDS-PAGE gel, we wanted to ensure the signal in the autoradiogram was due to WOX5 phosphorylation and *not* sACR4 autophosphorylation. Therefore, after incubation, the phosphorylated mWOX5 protein was pulled-down by amylose resin and extensively washed to remove autophosphorylated sACR4. Indeed, the appearance of the radioactive band was due to mWOX5 phosphorylation, as seen by the absence of sACR4 when incubated alone (Fig. 8, *lane 1*). A separate control reaction performed under the same conditions with sACR4 and MBP confirmed that the phosphorylation was specific towards WOX5 and not the MBP solubility tag (Fig.8, *lanes 5–7*).

**Fig 9:**
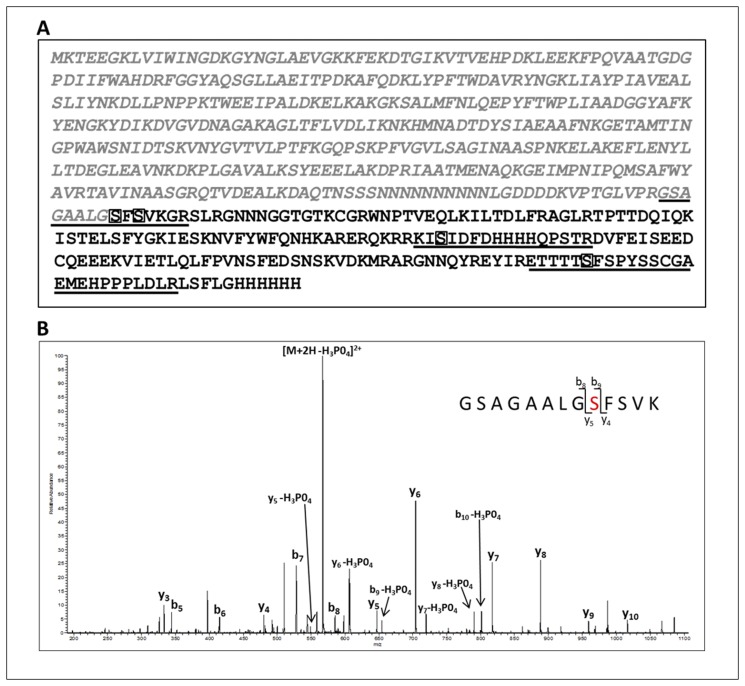
Identification of *in vitro* WOX5 phosphorylation sites. **(A)** The sequence of the MBP fused WOX5 protein is shown: the N-terminal MBP fusion tag (grey italics) and the 182aa WOX5 sequence with the C-terminal 6X-His tag (black). Note, the N-terminal Met residue of WOX5 was changed to Gly. Confirmed phosphorylation sites, Ser^2^, Ser^4^, Ser^88^, and Ser^158^ are boxed. The phosphopeptides identified by mass spectrometry are underlined. **(B)** Collision-induced low resolution fragmentation spectrum of the phosphopeptide GSAGAALGSFSVK, encompassing the C-terminal end of the fusion tag and the N-terminal end of WOX5. The presence of the phosphorylated b_9_ (theoretical *m/z* 654.32, observed *m/z* of 654.33) and y_5_ (theoretical *m/z* 549.30, observed *m/z* of 549.33) ions, and the unphosphorylated b_8_ (theoretical *m/z* 585.30, observed *m/z* of 585.17) and y_4_ (theoretical *m/z* 480.28, observed *m/z* of 480.30) ions, confirms Ser^2^ as the site of phosphorylation.

We next determined the sites of phosphorylation within the WOX5 protein by collision induced dissociation (CID) tandem mass spectrometry. The data dependent analysis of tryptic peptides and a search against the mWOX5 protein sequence (Fig. 9A) yielded several phosphorylated peptides. Assignment of the phosphorylation site was performed by verifying the presence of diagnostic site-discriminating *y* and *b* series ions. For example, the CID tandem mass spectrum of the phosphopeptide encompassing the junction between the C-terminal end of the solubility tag and the N-terminal sequence of WOX5, GSAGAALG(pS)FSVK, is shown in Fig. 9B. The predominant species is the [M+2H-98]^2+^ ion (*m/z* of 567.3) carrying a +2 charge state. This is characteristic of a neutral loss of phosphoric acid which is indicative of a phosphorylated peptide. Exact assignment of the phosphorylation at Ser2 (in WOX5 amino acid sequence) was verified by the presence of site-discriminating ions including the phosphorylated b_9_ (theoretical *m/z* 654.32, observed *m/z* of 654.33) and y_5_ (theoretical *m/z* 549.30, observed *m/z* of 549.33) ions and the unphosphorylated b_8_ (theoretical *m/z* 585.30, observed *m/z* of 585.17) and y_4_ (theoretical *m/z* 480.28, observed *m/z* of 480.30) ion. The absence of the phosphorylated b series of ions corresponding to b_2_-b_8_ and y series ions y_3_-y_4_, further confirmed that phosphorylation occurs at Ser2. A total of four phosphorylation sites were identified within the WOX5 sequence (Fig. 9A). Table 1 summarizes the site-discriminating ions for the four confirmed sites at Ser2, Ser4, Ser88, and Ser158 of the WOX5 protein. Intriguingly, phosphorylation at two Ser residues occurs in a ‘GSFS’ motif within WOX5. ACR4 also contains a ‘GSFS’ sequence located within the glycine-rich loop of the kinase structure and is autophosphorylated at the first Ser [19]. Furthermore, a third phosphorylation site in WOX5, Ser^158^, is found in a ‘TSFS’ motif that is highly similar to the ‘GSFS’ sequence. These data suggest a potential recognition motif for ACR4 substrate phosphorylation of WOX5 *in vivo*.

**Table 1:** Site-discriminating ions in WOX5 MS (the percentage of maximal intensity for each site-discriminating ion in the averaged low-resolution CID spectrum is shown in parenthesis.

Peptide	Residue Position in WOX5	b ion	Phosphorylated b ion	y ion	Phosphorylated y ion
**GSAGAALGSFSVK**	**2**	**b8 (+1, 6.45)**	**b9 (+1, 4.42)**	**y4 (+1, 6.50)**	**y5 (+2.41)**
**GSAGAALGSFSVKGR**	**4**	**b10 (+1, 5.22)**	b11 (—) *	**y4 (+2, 2.54)**	**y5 (+2, 1.79)**
**KISIDFDHHHHQPSTR**	**88**	**b2 (+1, 0.40)**	**b3(+1, 0.92)**	**y13 (+2, 1.30)**	**y14 (+2, 3.74)**
**ETTTTSFSPYSSCGAEMEHPPPLDLR**	**158**	**b5 (+1, 2.50)**	b6 (—) *	**y20 (+2, 16.59)**	**y21 (+2, 4.06)**

*supporting ion information is available)

## Discussion

In higher animals and plants, directed cell proliferation, specification, and differentiation during growth and development are guided by a variety of external stimuli that are perceived and interpreted by a multitude of cell surface receptors primarily mediated through RTKs in animals and by RLKs in plants. The accepted paradigm of signal transduction involves ligand perception/binding to the extracellular domain, activation of the intracellular kinase domain and subsequent activation of downstream signaling pathways through reversible phosphorylation events. Subsequently, downstream signaling components influence transcriptional activity in the nucleus allowing appropriate cellular responses to specific signaling cues.

In recent years, however, it has become apparent that the concept of a single receptor-single ligand interaction is perhaps too simplistic. Thus, structural, biophysical, biochemical and functional studies of several RTKs such as the Epidermal Growth Factor Receptor (EGFR) family [10–12, 44], Somatostatin and Opioid receptors [45], Insulin-like Growth Factor Receptor [46], and Platelet-derived Growth Factor (PDGF) [47] have demonstrated the capacity of these RTKs to assemble as homo- or heterodimeric complexes with distinct ligand specificity/affinities and downstream signaling activity. Similar heteromeric interactions occur among RLKs such as FLS2 and BAK1 [48], CLAVATA2 [49], and BRI1 [13–14] and their impact on downstream signaling cascades is well established. Importantly, in a recent study Stahl et al [24] have provided evidence from in planta studies that CLV1, an RLK essential for shoot stemness maintenance, can also moderate root stemness in *Arabidopsis* through homo- and heteromeric complexes with ACR4. This study not only documented the overlapping expression patterns of the two RLKs in the distal root meristem using reporter lines but unequivocally demonstrated their association with the plasmodesmata. Significantly, it was demonstrated through a split-luciferase assay and FRET-FLIM analysis that direct molecular interactions between ACR4 and CLV1 occurred at the plasmamembrane and was mediated through the transmembrane domains.

Among the five members of the ACR4 receptor family, limited genetic and cell biology analyses have suggested these receptors may act in the same genetic pathway through functional redundancy based upon gene duplication and/or through activation of signaling cascades via receptor heterodimerization [15, 17, 20]. RT-PCR studies of ACR4 and CRR transcript levels in varying tissues encompassing the entire *Arabidopsis* plant demonstrate all family members are expressed within all tissues examined, except for CRR2 and CRK1 which are not expressed in the siliques [17]. However, since physically interacting proteins must be spatiotemporally co-expressed in the same milieu to have any biological relevance, gene expression analysis often serves as a proxy for protein expression [50]. In the absence of reporter lines to experimentally demonstrate overlapping protein expression, we used two *in silico* gene expression data sets to compare the mRNA levels of members of the ACR4 family in vegetative tissue and roots. Thus, analysis of the microarray data for various tissues/cell types in Arabidopsis at the *Arabidopsis* “electronic fluorescent pictograph” (eFP) browser (http://bar.utoronto.ca) [51], very clearly indicated overlapping expression patterns for this family in the shoot apical meristem (SAM), stem epidermis (top of stem) and stigma/ovaries. Within the SAM, all five genes (ACR4, At3g59420; AtCRR1, At3g09780; AtCRR2, At2g39180; AtCRR3, At3g55950; AtCRK1, At5g47850) were expressed in the rib meristem and the peripheral zone with mRNA expression levels in the order ACR4>AtCRR2>AtCRR1>AtCRR3>AtCRK1. Interestingly, however, in the central zone there appeared to be very little expression of AtCRK1 although the expression levels of the other 4 genes followed the same order. A similar expression profile was observed in the stem epidermis (top of stem) and stigma/ovaries, but with non-detectable mRNA for AtCRK1 (data not shown). In analogous fashion we applied the co-expression analysis tool within CORNET 2.0 (https://cornet.psb.ugent.be/) [52, 53] specifically with the data set for expression in root. It is both significant and interesting that all five genes showed overlapping and comparable expression levels in the quiescent center, lateral root cap, root tip, root cortex and columella root cap cell (data not shown). Therefore, existing data showing overlapping tissue-level expression patterns leave open the possibility that the proteins could be co-expressed in the same cells and interact *in vivo* towards defined biological pathways.

In this study we have analyzed the *in vitro* interactions between the ICDs of ACR4 and the CRRs to elucidate the molecular aspects of protein-protein interactions among the kinase domains of this receptor family. Ideally, crystal structure determination of complexes between ACR4 and the CRRs would facilitate an atomistic level understanding of these interactions. In the absence of structural information, we utilized hydrodynamic analyses, peptide binding studies and pull-down assays to demonstrate protein-protein interactions among members of the ACR4 family. Gel filtration studies and pull-downs show that, in the unphosphorylated state, the ICD of ACR4 can self-associate (Fig. 1A and Fig. 2A, *lane 1*) and also bind to all four CRR ICDs (Fig. 1B and D and Fig. 2A), thus indicating that the intracellular regions of these receptors have the potential to form inactive heteromeric complexes. Indeed, examples exist in which RTKs are capable of preformed, inactive heterodimer complexes in a ligand independent manner. For instance, the EGFRs are able to form homo- or heterodimeric structures via their kinase domains in the absence of external signaling molecules [54–58]. Among RLKs, precedent for a heteromeric interaction exists in the five member AtSERK family, wherein AtSERK1 is able to heterodimerize with AtSERK2 in cowpea protoplasts [59]. Interestingly, the ICDs of CRR2, CRR3, and CRK1 do not show evidence of homodimerization (Fig. 1B, C, and D, *right panels*) although it is possible that they may have the capacity to form heteromers amongst themselves. Overall, given the ACR4-CLV1 interaction, it will be particularly interesting to see if the paradigm of heteromeric interactions holds for the ACR4 homologs as well. Such focused studies on the molecular basis of protein-protein interactions using *in vitro* biophysical/biochemical systems can inform on formulating new hypothesis-driven biological investigations.

We have also demonstrated that the ICD of ACR4 is capable of binding to the kinase-dead CRR1 and CRR2 ICDs regardless of the phosphorylation status (Fig. 2A-D). This observation is particularly interesting in light of the fact that in animals there are atypical RTKs with kinase-inactive domains that signal through phosphorylation-independent mechanisms [60]. A classic example is the ErbB3 receptor in the EGFR family that has a weakly active or non-functional kinase domain but is nevertheless a critical component of the EGFR signaling mechanism since it can form heterodimers with other members of the family that phosphorylate it [12, 61]. Atypical RLKs, functioning in an analogous manner, have also been reported in plants. Sequence analysis suggests that ~20% of the >600 RLKs encoded in the *Arabidopsis* genome are inactive, atypical RLKs, and it is likely that activation of differential signaling pathways can occur through heterodimerization among this RLK subfamily [34, 62–63]. Therefore, it is quite possible for ACR4 to expand its signaling cascade through interactions with the kinase-dead homologs.

Our *in vitro* kinase assays demonstrate that active ACR4 kinase domain can phosphorylate the ICDs of all the CRR homologs, therefore suggesting that ACR4 signaling *in vivo* could potentially occur through heterodimerization with CRR family members. Similar mechanisms have been determined in plant RLKs in which cytoplasmic domains in heterodimeric receptor complexes can undergo *trans* phosphorylation [31, 64–65]. Importantly, binding of different ligands to the ACR4 extracellular domain may also promote preferred heterodimerization with CRR family members to initiate a diverse array of signaling pathways that could alter signaling output and duration. For instance, members of the EGF receptor family can bind a variety of ligands which promotes homo- or heterodimerization that can activate multiple downstream signaling cascades [10, 12]. Although one peptide, CLE40, has been proposed to be the ligand for ACR4 [21–22], it does not preclude the possibility that other ligands may act in the ACR4 signaling pathway. Indeed, there are 32 members of the CLE peptide family in *Arabidopsis* that affect developmental functions in the plant [66,67]. However, the role of these presumed peptide ligands in ACR4 or CRR signaling has yet to be determined.

Both intramolecular and intermolecular protein-protein interactions are driven by contacts between specific residues on the interfacial faces of the participating domains. In contrast to stable complexes, biological signal transduction is regulated in large part by transiently formed states in which the proteins may switch between bound and unbound forms through modification-dependent conformational changes. While it is not possible to accurately define the state of the ACR4/AtCRR interactions based on current data, nevertheless our peptide-binding and HD exchange experiments indicate the importance of the N-lobe region of ACR4 kinase domain in heteromeric interactions. Specifically, our results suggest that the KDSAF motif in CRR3/CRK1 is important for contacts to the LLSLL region in ACR4 ICD (Fig. 6) and is further supported by the fact that this binding is significantly reduced when these residues are mutated to alanines (Fig. 8). Furthermore, our HDX studies indicate protection of solvent accessible region FRTELDL, which is in close proximity to the LLSLL region at the helix-C of ACR4 ICD in a 3D Model (data not shown). Interestingly, in the case of the RTKs, the involvement of the N-lobe in modulation of kinase activity, intermolecular and intramolecular interactions is well documented [1, 68–70, 71, 72].

It appears that ACR4 is involved in at least two distinct signaling pathways with overlapping themes, stem cell differentiation in the root tip and differentiation of the epidermis in vegetative and reproductive tissue. As a mediator of stem cell differentiation in the root tip (20–22), ACR4 is described to perceive the extracellular CLE40 peptide ligand which promotes stem cell differentiation. Presumed activation of ACR4 then influences the expression domain of the WOX5 transcription factor, a protein that promotes stem cell proliferation. Our experiments have demonstrated that the ACR4 kinase can interact with and phosphorylate WOX5 *in vitro*. At this time, the relevance of these phosphorylation sites to the *in vivo* function of WOX5 is not known. Complementary *in vivo* experiments knocking out the potential phosphorylation sites in the WOX5 protein may drive phenotypic effects in transformed *wox5* mutant plants aiding in the characterization of the functional significance of these phosphorylated residues. However, although WOX5 is deemed to be a key player in root stem cell regulation, it would be presumptuous to ignore the parallel inputs derived from other transcription factors [73] or potential regulation influenced by the combinatorial activity of different transcription factors [74].
